# Strategies to improve patient-reported outcome completion rates in longitudinal studies

**DOI:** 10.1007/s11136-019-02304-8

**Published:** 2019-09-23

**Authors:** Lene Kongsgaard Nielsen, Madeleine King, Sören Möller, Mary Jarden, Christen Lykkegaard Andersen, Henrik Frederiksen, Henrik Gregersen, Anja Klostergaard, Morten Saaby Steffensen, Per Trøllund Pedersen, Maja Hinge, Mikael Frederiksen, Bo Amdi Jensen, Carsten Helleberg, Anne Kærsgaard Mylin, Niels Abildgaard

**Affiliations:** 1 Department of Haematology, Quality of Life Research Center, Odense University Hospital, Odense, Denmark; 2 OPEN, Odense Patient Data Explorative Network, Odense University Hospital, Odense, Denmark; 3 Faculty of Science, School of Psychology, University of Sydney, Sydney, Australia; 4 Department of Clinical Research, University of Southern Denmark, Odense, Denmark; 5 Department of Haematology, Copenhagen University Hospital, Copenhagen, Denmark; 6 Department of Haematology, Aalborg University Hospital, Aalborg, Denmark; 7 Department of Haematology, Aarhus University Hospital, Aarhus, Denmark; 8 Department of Haematology, Regional Hospital West Jutland, Holstebro, Denmark; 9 Department of Haematology, South West Jutland Hospital, Esbjerg, Denmark; 10 Department of Internal Medicine, Vejle Hospital, Vejle, Denmark; 11 Department of Haematology, Hospital of Southern Jutland, Aabenraa, Denmark; 12 Department of Haematology, Zealand University Hospital, Roskilde, Denmark; 13 Department of Haematology, Herlev Hospital, Herlev, Denmark; 14 The Academy of Geriatric Cancer Research (AgeCare), Odense University Hospital, Odense, Denmark

**Keywords:** Missing data, Health-related quality of life, Patient-reported outcomes, Patient-reported outcomes completion rate, Multiple myeloma

## Abstract

**Purpose:**

The quality of patient-reported outcome (PRO) data can be compromised by non-response (NR) to scheduled questionnaires, particularly if reasons for NR are related to health problems, which may lead to unintended bias. The aim was to investigate whether electronic reminders and real-time monitoring improve PRO completion rate.

**Methods:**

The population-based study “Quality of life in Danish multiple myeloma patients” is a longitudinal, multicentre study with consecutive inclusion of treatment-demanding newly diagnosed or relapsed patients with multiple myeloma. Education of study nurses in the avoidance of NR, electronic reminders, 7-day response windows and real-time monitoring of NR were integrated in the study. Patients complete PRO assessments at study entry and at 12 follow-up time points using electronic or paper questionnaires. The effect of the electronic reminders and real-time monitoring were investigated by comparison of proportions of completed questionnaires before and after each intervention.

**Results:**

The first 271 included patients were analysed; of those, 249 (85%) chose electronic questionnaires. Eighty-four percent of the 1441 scheduled PRO assessments were completed within the 7-day response window and 11% after real-time monitoring, achieving a final PRO completion rate of 95%. A significant higher proportion of uncompleted questionnaires were completed after the patients had received the electronic reminder and after real-time monitoring.

**Conclusions:**

Electronic reminders and real-time monitoring contributed to a very high completion rate in the study. To increase the quality of PRO data, we propose integrating these strategies in PRO studies, however highlighting that an increase in staff resources is required for implementation.

## Introduction

Multiple myeloma (MM) is an incurable malignancy of plasma cells in the bone marrow. MM is associated with severe morbidity, specifically caused by bone destruction and pathological bone fractures, renal dysfunction, high infection rate and potential physical disability [1, 2]. The prognosis of MM has improved markedly over the past 20 years, and the median survival of MM patients under the age of 70 has increased from 3 years [3] to 6–7 years [4, 5]. The improved prognosis is mediated by the introduction of high dose chemotherapy with autologous stem cell support (HDT) in the 1990s, new treatment options with immunomodulatory drugs (IMiDs), such as thalidomide, lenalidomide and pomalidomide [6–8], and the proteasome inhibitors, bortezomib, carfilzomib and ixazomib [9–11]. Most recently, the monoclonal antibodies elotuzumab and daratumumab [12, 13] have been introduced, and therefore the prognosis is expected to improve even further in coming years [14].

Treatment choice in MM depends on the patients’ age, disease complications, existing comorbidity and whether the patient is judged fit for specific regimens, such as HDT. Treatment usually involves repeated cycles of a 2–3 drug combination therapy with a proteasome inhibitor, IMiD, cytostatic agent and/or steroid. Treatment implies a risk of both acute adverse events, such as infections, as well as late effects, such as peripheral neuropathy and fatigue [15–18].

Patients with MM report a high symptom burden. Common symptoms include fatigue, pain, constipation, insomnia and tingling in the hands/feet, with a consequent decrease in physical and cognitive functioning [19–21]. Compared to patients with other haematological malignancies, patients with MM report low health-related quality of life (HRQoL) [21, 22]. Longitudinal HRQoL studies of patients with MM suggest that clinically beneficial improvements in HRQoL are more likely during primary treatments than during treatment for relapse [23].

The patients’ experience of symptoms and HRQoL can be validly and reliably captured with patient-reported outcome (PRO) questionnaires [24]. Integration of PROs into routine care management of patients with advanced cancer is associated with increased overall survival and reduction of the symptom burden [25, 26]. Typically, PRO assessments are scheduled at key time points and when a questionnaire is not completed at a scheduled time, it is referred to as a non-response (NR). Consequently, missing data can lead to a variety of problems, more so as NR rates increase, including loss of study power and precision [27, 28]. Further, there is risk of bias if the reason for NR is related to the patient’s health status and appropriate analysis methods are not applied. For example, if only complete case analysis methods are used, there is a risk of overestimated HRQoL and underestimated toxicity [27–29]. This is due to the analysis being based on available data from patients with completed PRO assessments and who presumably have better HRQoL, while patients who drop out might have more toxicity and a worse HRQoL [27, 30–34]. Thus, NR represents a threat to internal and external validity and is one of the inherent barriers in establishing high quality PRO data for use in patient-centred care [35–37]. Several strategies designed to minimize NR have been proposed, and these can be integrated into the study design, protocol and implementation procedures for the PRO study [29]. These include ensuring that staff are aware of the importance of reducing NR and have access to written study procedure guidelines and support [29, 31]. Also, given the time-sensitive nature of PRO data, real-time monitoring of PRO completion rates during the study is recommended [38]. However, we are not aware of any studies that have assessed and documented the effectiveness of such strategies in reducing NR.

The study “Quality of life in Danish multiple myeloma patients” (QoL-MM) is a national multicentre, prospective, observational and primarily web-based survey with real-time monitoring of NRs. Several strategies to reduce NR have been implemented including education of study nurses, electronic reminders and real-time monitoring of PRO completion. The aim of this analysis was to investigate whether electronic reminders and real-time monitoring of NRs improve PRO completion rate.

## Methods

The QoL-MM study includes newly diagnosed or relapsed, treatment-demanding patients with MM who, according to International Myeloma Working Group (IMWG) criteria, are eligible for inclusion [2]. Broad inclusion criteria ensure inclusion of a population-based, representative cohort of MM patients. Only, patients who are not able to understand the Danish language or who are diagnosed with a psychiatric condition are ineligible. Study sites include all 10 Danish departments of haematology. The goal is to recruit 800 patients, and each patient is followed for 24 months or until early drop-out due to withdrawal of consent, death or permanent lack of ability to fill out the questionnaires. The patients are introduced to the study by their treating physician or nurse, and written informed consent is obtained prior to inclusion. Demographic data are collected as part of the inclusion interview by a local study nurse. Moreover, the patients provide information related to activity of daily living (ADL), instrumental ADL and self-reported diseases, summarized into the Charlson Comorbidity Index [39–41]. This information is used to calculate the Freiburger Comorbidity Index of 0-3 and the IMWG myeloma frailty score which divides the patients into three categories of “Frail”, “Intermediate Fitness” or “Fit” [42, 43]. The patient’s Karnofsky Performance status was assessed by the local study nurse [44]. Clinical data, e.g. date of diagnosis, MM subtype and the prognostic score, International Staging System, are collected from The Danish Multiple Myeloma Registry [45, 46]. Data on admissions, discharges and other hospital procedures are captured from The National Registry of Patients [47].

### PRO study design

Patients complete questionnaires at study entry and at 12 follow-up time points during a 24 month period. The patient left the study before 24 months in case of death, withdrawn consent or entering a state with permanently inability to complete a questionnaire. The follow-up target dates for completion of questionnaires are every 4 weeks for the first 6 months and thereafter every 3 months until 24 months. Depending on the PRO assessment time point, the patient completes between two and four PRO instruments, equivalent to 50–85 items. Each set of questionnaires is sequenced to begin with the cancer specific instrument European Organisation for Research and Treatment of Cancer Quality of life QLQ-C30 (EORTC QLQ-C30) [24] followed by the Multiple Myeloma module QLQ-MY20 (EORTC QLQ-MY20) [48], the Chemotherapy-Induced Peripheral Neuropathy module (EORTC QLQ-CIPN20) [49] and the Short-form health survey version 2 (SF12v2) [50].

### PRO data collection procedures

Participants are asked to complete the entire set of questionnaires at one time, preferably on the target date and no later than 7 days hereafter, which is the 7-day response window for all follow-up questionnaires. The patients are furthermore encouraged to use the web-based questionnaire method, where a link is sent to the patients’ email on the target date. However, patients can choose the paper-and-pencil method, if preferred. A REDCap database automatically sends the emails on the target date and email reminders [51].

At baseline, the patient may complete the questionnaires alone or with assistance from the study nurse using a tablet or paper. The baseline questionnaires can also be completed at home by computer or tablet; however the study nurse must ensure that the questionnaires have been completed no later than on the day of anti-myeloma treatment. The patient is excluded as a screening failure if there is an uncompleted baseline questionnaire or missed completion of one or more of the four baseline PRO instruments by 3 days after start of anti-myeloma treatment.

Patients who choose to use paper questionnaires receive three sets of questionnaires at the inclusion interview to complete at four, eight and 12 weeks follow-up. The target date of each set of questionnaires is written on the cover page with the contact information of the local study nurse. The patients are asked to bring the completed questionnaires to the outpatient clinic at a scheduled appointment. To remind the patient to complete the first follow-up paper questionnaire, the local study nurse contacts the patient at 4 weeks. After week 12, it is the responsibility of the local study nurses to provide the next three sets of questionnaires to the patient for completion at 16 and 20 weeks and 6 months. In this way, the local study nurse provides paper questionnaires to the patients who chose to complete the follow-up questionnaires in paper, three times during the 24 months. Completed paper questionnaires are uploaded to the REDCap database by the local study nurse and centrally entered continuously during the study period by the staff at the study office.

### Strategies to minimize intermittent non-responses

All local study nurses are informed about the importance of minimizing missing items and NR to a questionnaire or set of questionnaires and are trained in appropriate procedures to minimize missing data. Local study nurses are permitted to provide support to frail patients or patients who temporarily lack the ability to independently complete questionnaires. In such cases, the local study nurse reads the items and response categories aloud and marks the patient’s answers on his or her behalf. The aim is for this to be done within the 7-day response window after the target date. If the patient does not have an appointment in the outpatient clinic within this time frame, but has a scheduled appointment a few days before the target date, the study nurse provides the patient with the questionnaire at that appointment. Otherwise, the questionnaire is completed after the 7-day response window.

For patients using the web-based completion method who have not completed the electronic questionnaire at day 4, a reminder is automatically sent to the patients email. If a patient has still not answered the questionnaire on day 7 after the target date, the local study nurse is notified by the central study office during week days, as part of real-time monitoring of NR. In this situation, the local study nurse has 2 week days to contact the patient, ascertain and document the reason for NR and to invite the patient to complete the questionnaire. The study nurses have access to a written guideline of all project related tasks, and in case there is need for further clarification of a project procedure, the study office can be contacted by telephone or email during week days.

Real-time monitoring of PRO completion or NR for both web-based and paper-based PRO questionnaire completion is carried out by the study office. If a patient has completed some or all items of the EORTC QLQ-C30, which is the first questionnaire at every scheduled PRO assessment time point, the follow-up PRO assessment is defined as completed. Missing items and partly completed questionnaires or sets of questionnaires are not part of the real-time monitoring.

The participating departments are financially compensated for managing a NR, providing guidance to a patient to complete a questionnaire and when three completed paper questionnaires are either provided or collected.

### Information to the participants

As part of the inclusion interview, all patients are informed about the importance of completing the follow-up questionnaires within the 7-day response window, and that the study nurse will contact them if they have not completed a questionnaire by the seventh day. A part of the information provided to the patients choosing the electronic platform is that they will receive an email reminder, if they have not completed a scheduled questionnaire within 4 days. The patients are informed about the reason why they will be contacted in case of an NR within the 7-day response window, including that this could be due to a decline in the patients’ HRQoL. Patients are informed that participation and completing questionnaires in the study are voluntary and that choosing to skip a scheduled questionnaire will not have any consequences for the patient. All patients receive the study nurses’ contact information and are encouraged to seek support in case of questions, technical challenges or deciding to change the method of completion.

### Patient cohort and data analysis

The patient cohort for this paper include all patients who consented to QoL-MM by 16th August 2018 and who reached at least the first follow-up PRO assessment time point at week 4. Questionnaires which are completed before or within the 7-day response window are defined as “on-time responses”. In case the patient complete the questionnaire at day 7 after the target date or later, the response is defined as “salvage response”, and the remainder were categorized as a “never response”. The PRO completion rate was calculated as the number of completed on-time and salvage responses as a proportion of the number of scheduled PRO assessments expected to be completed [52]. Also, we calculated the rate of on-time and salvage responses. The effect of electronic reminders was estimated by comparing the number of completed questionnaires completed by patients who had chosen to complete the follow-up questionnaires electronically at day four compared to day three in relation to the number of questionnaires still not completed. The same was carried out to estimate the effect of real-time monitoring, where the number of electronic completed questionnaires at day 6 and day 7 was compared. Chi square test was used as statistical analysis method for the effect analyses. We investigated seven baseline patient characteristics as predictors for on-time response and questionnaire completion (age, gender, Karnofsky Performance Status Scale, IMWG frailty score, CCI, relapsed disease and completion of the questionnaires by paper). The analyses were performed by mixed effects logistic regression with a random effect to take account non-independence of observations from the same patient. *P*-values < 0.05 were considered statistically significant. Data are presented by descriptive analyses using Stata version 15.

## Results

As of August 16th 2018, 481 patients were found eligible for the QoL-MM study, and hereof 292 provided written consent for participation and inclusion in the study. Of the 292 patients included, 271 had reached at least the first follow-up PRO assessment time point at 4 weeks and were included in the analyses. Patient and disease characteristics are presented in Table 1. Of all patients, 55% had newly diagnosed symptomatic MM of which 52% started an induction regimen with planned HDT at study entry. A daratumumab containing regimen was the most frequently used regimen in treatment of relapse MM and 24% of the patients with relapse started fifth or later line of therapy. Twenty-four percent of both newly diagnosed and relapse MM were 76 years or older, and 17% of the NDMM patients and 12% of the RMM were characterized as “Frail” according to the IMWG myeloma frailty score.

**Table 1:** Demographic and disease characteristics at entry

Characteristics	Newly diagnosed patients with MM *P* = 165	Relapse patients with MM *P* = 127
Mean age (SD)	67.0 (9.7)	68.7 (9.5)
Median age, years (IQR)	68 (60; 74)	70 (63; 74)
Age ≤ 65/66–75/≥ 76 years, N (%)	56 (34%)/69 (52%)/40 (24%)	40 (32%)/57 (45%)/30 (24%)
Sex, female/male, *N* (%)	70 (42%)/95 (58%)	42 (33%)/85 (67%)
Marital status, married or cohabiting/single^a^, *N* (%)	131 (79%)/34 (21%)	99 (78%)/28 (22%)
Weekly alcohol intake, no alcohol intake/1–7/> 8 items, *N* (%)	42 (25%)/81 (49%) 42 (25%)	24 (19%)/70 (55%)/33 (26%)
Daily smoking, yes/former smoker/never smoker, *N* (%)	19 (12%)/75 (45%) 71 (43%)	15 (12%)/57 (45%)/55 (43%)
Charlson Comorbidity Index, 0/1/2/≥ 3, *N* (%)	100 (61%)/20 (12%)/27 (16%) 18 (11%)	66 (52%)/28 (22%)/21 (17%)/12 (9%)
Freiburg Comorbidity Index, 0/1/2 or 3, *N* (%)	124 (75%)/40 (24%)/1 (1%)	109 (86%)/17 (13%)/1 (1%)
IMWG Myeloma Frailty Score, Fit/intermediate fitness/Frail, *N* (%)	82 (50%)/55 (33%)/28 (17%)	77 (61%)/35 (28%)/15 (12%)
Karnofsky Performance Status Scale, 100/90/80/**≤ **70%, *N* (%)	55 (33%)/54 (33%)/30 (18%)/26 (16%)	28 (22%)/65 (51%)/23 (18%)/11 (9%)
Mean time from diagnosis to inclusion (years) (SD)	0.16 (0.77)	4.69 (3.05)
M-component subtype, IgG/IgA/light chain/> 1 M-component/non-secretory/missing^b^	28 (63%)/11 (25%)/3 (7%)/0/2 (5%)/121	59 (63%)/21 (22%)/8 (9%)/2 (2%)/4 (4%)/33
International Staging System, ISS I/ISS II/ISS III/missing^b^, *N* (%)	13 (30%)/19 (44%)/11 (26%)/122	11 (19%)/34 (59%)/13 (22%)/69
Number of lines of therapy		
First line	165 (100%)	–
Second line	–	50 (39%)
3–4 line	–	47 (37%)
5 or more lines	–	30 (24%)
Anti-myeloma treatment^c^		
Induction therapy and HDT	86 (52%)	8 (6%)
Melphalan-prednisolon-bortezomib	47 (28%)	3 (2%)
Containing daratumumab	0	70 (55%)
Containing elotuzumab	0	7 (6%)
Lenalidomide	6 (4%)	8 (6%)
Containing Ixazomib	1 (1%)	7 (6%)
Containing carfilzomib	0	15 (12%)
Containing pomalidomide	1 (1%)	6 (5%)

*P* number of patients, *SD* standard deviation, *IQR* interquartile range, *IMWG* International Myeloma Working Group, *HDT* high dose therapy with stem cell support, *ISS* International Staging System

^a^Separated, divorced, widow or unmarried

^b^Missings are due to time delay in entering disease data into The Danish National Multiple Myeloma Registry or unknown

^c^Glucocorticosteroid treatment is not registered in The National Registry of Patients

Electronic completion of follow-up questionnaires was chosen by 85% of the 271 patients, and 15% chose the paper-and-pencil method. Three patients changed mode of answering during follow-up, two from electronic method to paper method, as the electronic method was found to be too complicated.

### PRO completion rate

**Fig. 1:**
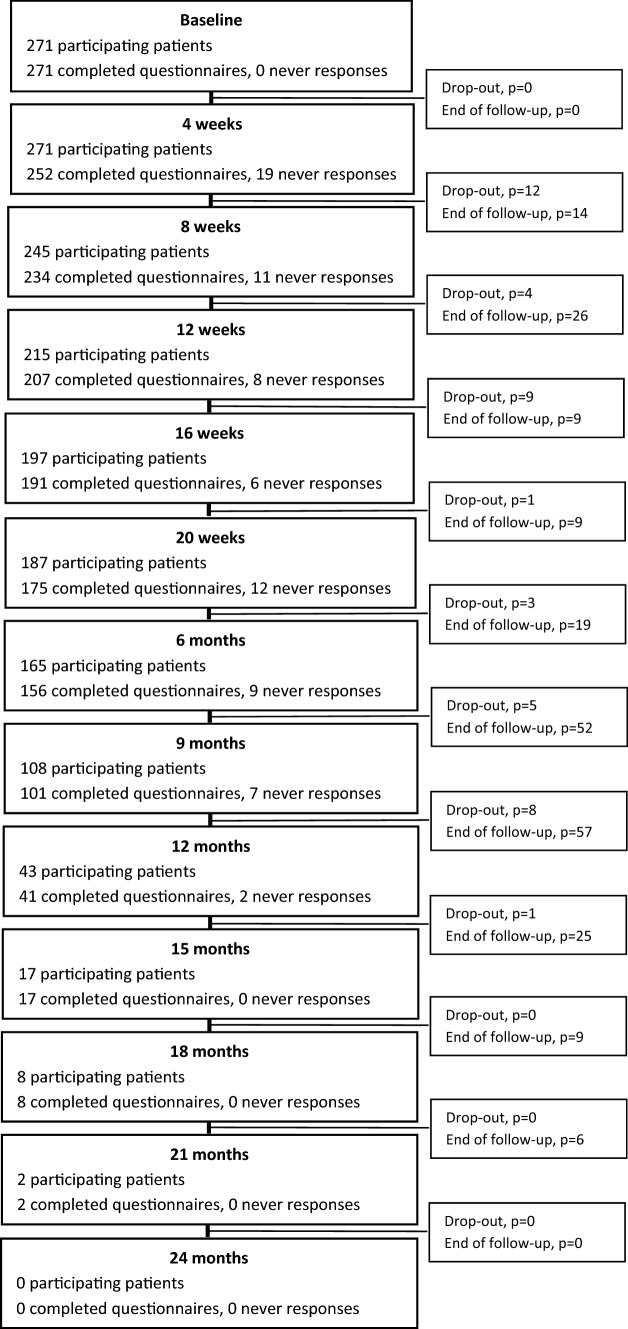
Flow diagram of the patients in follow-up. The reduction of patients in follow-up was due to drop-out or end of follow-up

Per protocol, for the study cohort (*n* = 271), 1441 scheduled follow-up questionnaires were expected to be completed at the time of analysis. The number of patients and completed questionnaires (on-time and salvage) and never-responses at each follow-up time point are presented in Fig. 1. The reasons for reduction in number of patients in the flow diagram are due to early drop-out or end of follow-up. The largest proportion of never-responses with the first year of follow-up was at 4 weeks 7% (19 questionnaires out of 271 expected).

Of the 1441 scheduled questionnaires, 1214 (84%) were completed on-time. Of the 227 questionnaires that were not completed on-time, 153 (67%) were salvaged responses and 74 (33%) were never completed. When adding the salvage responses to the on-time responses, a total 1367 of the scheduled questionnaires were completed, equivalent to a PRO completion rate of 95%.

### Pattern of response and effect of electronic reminder and real-time monitoring

**Fig. 2:**
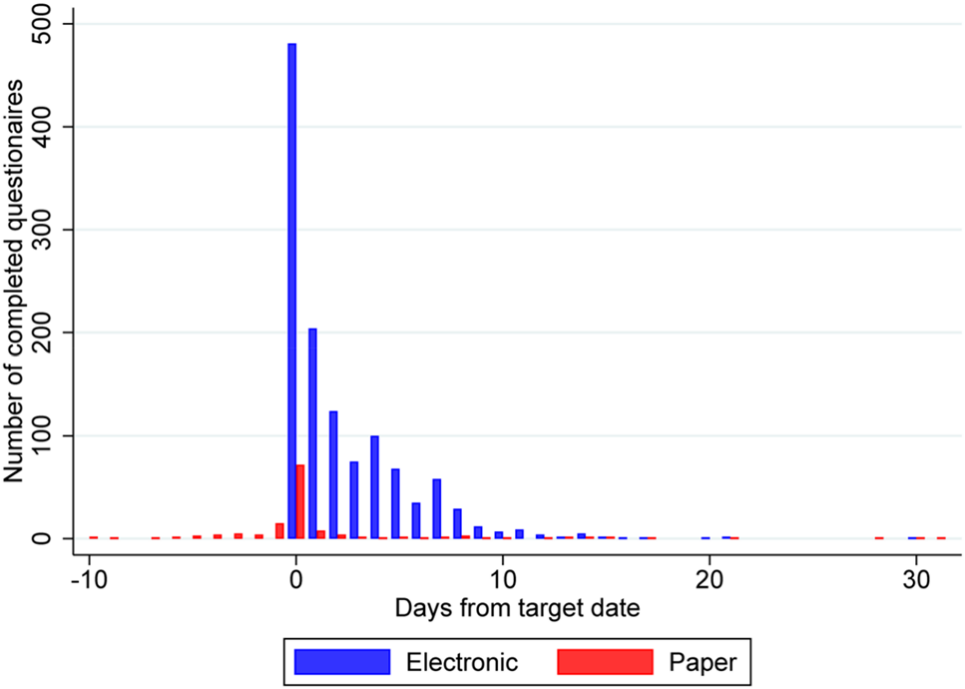
Day of response to paper and electronic questionnaires. Day 0 is the target day, when the patients were instructed to complete the questionnaires. The patients completing the questionnaires electronically received an email with a link to the questions on day 0. The local nurses provided the paper questionnaires with inscribed target dates for patients completing on paper to answer the questionnaires at home. If the patient had completed the EORTC QLQ-C30, which was the first health-related quality of life instrument in each set of questionnaires, the set of questionnaires was defined as completed

Questionnaire completion patterns are presented in Fig. 2; Table 2. Of the 1367 scheduled questionnaires, 553 (40%) were answered on the target date, 417 (31%) were completed on days one to three. For estimation of the effect of the reminder, at day 4, 100 out of 392 not completed electronic questionnaires were completed, which is 25.5% (95% CI 21.3%; 30.1%). At day 3, 75 out of 467 not completed questionnaires were completed, which is 16.1% (95% CI 12.8; 19.7). A significantly higher proportion of uncompleted questionnaires were completed after the patients had received the electronic reminder (*p* < 0.001). For estimation of the effect of real-time monitoring, at day 7, 58 out of 189 not completed questionnaires were completed (30.7%, 95% CI 24.2%; 37.8%). This is a significantly higher proportion (*p* < 0.001) than at day 6, where 35 out of 223 not completed questionnaires were completed (15.7%, 95% CI 11.2%; 21.1%).

**Table 2:** Pattern of response

Proportion of scheduled questionnaires	Time of response	Completed follow-up questionnaires (*Q *= 1367)
On-time response	Before day 0	37 (3%)
84%	Electronic	0
	Paper	37
	Day 0—the target day	553 (40%)
	Electronic	481
	Paper	72
	Day 1–3	417 (31%)
	Day 1	212
	Day 2	128
	Day 3	77
	Electronic	403
	Paper	14
	Day 4*–6	207 (15%)
	Day 4	101
	Day 5	70
	Day 6	36
	Electronic	203
	Paper	4
Salvage response	Day 7 or later	153 (11%)
11%	Day 7	60
	Day 8	32
	Day 9	13
	After day 9	48
	Electronic	134
	Paper	19

Questionnaires completed before or within the 7-day response window are termed “on-time responses”. Questionnaires completed after the 7-day response window are termed “salvage responses”. Five percent of the scheduled questionnaires were never completed

*If the patients completing questionnaires electronically did not respond by day 4, a reminder was sent by email

### Predictors for non-completion

The strongest predictors for non-completion of scheduled questionnaires were IMWG frailty score (Intermediate fitness; *p*-value 0.009, Frail; *p*-value 0.001) and choosing paper questionnaires (*p*-value 0.005). A weak association for non-completion was found for CCI 1 (*p*-value 0.042) and a weak association for completion was found for male gender (*p*-value 0.044). When we made the analysis for on-time completion only, the association between paper completion method and non-completion was persistent (*p*-value 0.047) and Karnofsky Performance Status Scale ≤ 70% (*p*-value 0.025) appeared as a predictor for non-completion, too. The results of the mixed effects logistic regression analysis are presented in Table 3. We repeated the analysis but adjusted for time point and found similar results (data not shown).

**Table 3:** Results of mixed effects logistic regression analysis

Predictor	Completed at all		Completed on-time	
	OR (95% CI)	*P*-value	OR (95% CI)	*P*-value
Age group				
< 65	1 (Reference)		1 (Reference)	
65–74	1.45 (0.41, 5.10)	0.560	1.11 (0.63, 1.95)	0.727
75+	4.27 (0.74, 24.56)	0.103	1.77 (0.75, 4.17)	0.191
Gender				
Female	1 (Reference)		1 (Reference)	
Male	**3.00 (1.03, 8.73)**	**0.044**	0.88 (0.53, 1.46)	0.611
Karnofsky Performance Status				
100%	1 (Reference)		1 (Reference)	
90%	0.77 (0.22, 2.75)	0.689	0.73 (0.39, 1.36)	0.315
80%	1.76 (0.35, 8.70)	0.490	0.49 (0.24, 1.00)	0.050
≤ 70%	1.54 (0.20, 11.87)	0.679	**0.35 (0.14, 0.88)**	**0.025**
IMWG Fraility Score				
Fit	1 (Reference)		1 (Reference)	
Intermediate Fitness	**0.11 (0.02, 0.57)**	**0.009**	0.53 (0.24, 1.19)	0.126
Frail	**0.02 (0.002, 0.21)**	**0.001**	0.40 (0.13, 1.19)	0.100
Charlson Comorbidity Index				
0	1 (Reference)		1 (Reference)	
1	**0.23 (0.06, 0.95)**	**0.042**	0.92 (0.46, 1.84)	0.806
2	2.31 (0.43, 12.55)	0.332	1.19 (0.49, 2.90)	0.703
3+	2.95 (0.42, 20.48)	0.274	1.77 (0.65, 4.81)	0.263
Relapsed disease				
No relapse	1 (Reference)		1 (Reference)	
Relapse	0.82 (0.28, 2.41)	0.712	0.76 (0.46, 1.25)	0.277
Questionnaire method				
Electronic	1 (Reference)		1 (Reference)	
Paper	**0.15 (0.04, 0.57)**	**0.005**	**0.48 (0.23, 0.99)**	**0.047**

Bold odd ratio and *P*-values represent statistically significant associations

*OR* odd ratio, *IMWG* International Myeloma Working Group

## Discussion

The aim of the analysis was to investigate whether the strategies used to minimize NRs to scheduled questionnaires could increase PRO completion rate in a longitudinal study of MM patients receiving anti-myeloma treatment. Our initiatives included education of study nurses, electronic reminders and real-time monitoring of NR. A significant higher proportion of uncompleted questionnaires were completed after the patients had received the electronic reminder and after real-time monitoring. Those strategies resulted in a very high PRO completion rate of 95%, with just 5% non-response.

PRO completion rates in longitudinal studies of patients with MM are often not reported, and of the studies, where this information was reported, the PRO completion rate was 78–98% [53]. Only one study has reported a higher PRO completion rate compared to our findings. This was the NMSG 4/90 study by Gulbrandsen et al. [54], where the PRO completion rate of the historical control group of newly diagnosed patients with MM was 98%. This historical control group originates from the EORTC QLQ-C30 validation study of patients with MM [55]. One of the aims in the validation study was to evaluate the applicability of the questionnaire in a cohort of patients with MM and included sampling of data concerning the patient’s need for assistance in completing the questionnaires. The authors found that up to 30% of the MM patients reported need of assistance in completing the questionnaire. Other strategies of how this high PRO completion rate was achieved are not described in the paper [55].

When the QoL-MM study was designed, there had been particular focus on how we could minimize NR. We introduced real-time monitoring of NR and provided the patients with reminders. Staff resources were dedicated for this purpose, software as well as a high proportion of the patients choosing the web-based questionnaire method made it possible, and we succeeded in reaching a high PRO completion rate. Still, some NR could not be avoided. We found that low performance status at the time of inclusion and choosing paper-and-pencil method were predictors for not completing the questionnaires on-time. Risk of never response was higher for females, un-fit patients at baseline and choosing paper method for questionnaire completion. Special attention and guidance should be provided from the study staff to avoid NR from those patients. Information about the clinical status of patients when they fail to complete a scheduled questionnaire and the reason for NR might assist the PRO researchers in making the correct assumption for the underlying missing data mechanisms [27, 28, 56]. The link between the documented reasons, the predictors for NR and the missing data mechanisms as well as estimation of the impact of NR and patient drop-out on HRQoL results will be investigated in future analyses of the QoL-MM study.

The overall aim of the QoL-MM study is to describe the quality of life of the general population of patients with MM from diagnosis to late, advanced disease throughout different kinds of anti-myeloma therapies [57]. Methodological considerations concerning PRO assessment time points were included as part of the study planning. Clinical visits are frequently chosen time points for PRO assessment in clinical trials of MM patients [53]. This decision has the advantage of reducing the risk of NR, since patients have the opportunity to complete the questionnaires at the hospital with assistance from the study nurse. Disadvantages in using day 1 of treatment cycles include a potential risk of underestimation of toxicities that occur after day 1 and not capturing periods with temporary decline in HRQoL, resulting in rescheduling of chemotherapy [58]. We chose to collect the PRO data in QoL-MM at predefined calendar time points to meet the overall study aim and thereby capture HRQoL at regular non-clinic time points throughout the MM patients´ diverse disease trajectories. This decision could have made the study vulnerable for low PRO completion rates, since the general population of patient with MM can be frail and are at risk of adverse events, hospital admissions, as well as risk of physical and mental disabilities caused by the disease and therapy. Therefore, we implemented the educational and procedural strategies to reduce NRs. The same strategies might be useful to ensure high PRO completion rate in routine assessment and clinical utilization of PRO measures in the broad group of cancer patients [59].

Another strategie we chose was to use a pre-planned time frame of 7 days for each scheduled questionnaire. This allowed the study nurses to clearly communicate the expectations to the patients participating in the study and systematically capture reasons for NR from every patient who failed to complete the questionnaire within the 7-day response window [60, 61]. Whether patients who were not able to complete the questionnaires within the expected time frame have a poorer HRQoL will be investigated as part of the QoL-MM study.

## Conclusions

We evaluated strategies to maximize PRO completion in a longitudinal cohort study of patients with MM receiving anti-myeloma treatment. Real-time monitoring of NR, electronic reminders and education of study nurses are effective strategies that resulted in a questionnaire completion rate of 95%. To our knowledge, the QoL-MM study is the first study to provide insight into how to ensure high PRO completion rates in a cohort of cancer patients receiving chemotherapy. We propose our applied strategies as a model for improving PRO completion rates in clinical trials, registries and routine care management to increase the quality and value of PRO data in patient-centred care.
